# The equilibrium point hypothesis revisited: why threshold control does not explain human movement

**DOI:** 10.1007/s00221-026-07242-9

**Published:** 2026-02-13

**Authors:** Madhur Mangalam, Nick Stergiou

**Affiliations:** 1 Department of Biomechanics, University of Nebraska at Omaha, Omaha, NE 68182 USA; 2 Department of Physical Education and Sport Science, Aristotle University, 570 01 Thessaloniki, Greece

**Keywords:** Motor control, Muscle mechanics, Neuromechanics, Computational homunculus, Theoretical critique, Neuroscience

## Abstract

The equilibrium point hypothesis (EPH) proposes that movement control arises from shifting referent configurations ($\lambda$) that set muscle activation thresholds, with behavior emerging from neuromechanical interactions. Here, we review theoretical, neurophysiological, and computational evidence indicating that, for realistic multi-joint behavior, EPH is under-specified: it does not resolve inverse kinematics/dynamics, impedance regulation, or temporal coordination, and it conflicts with established force–length and force–velocity muscle properties and task-dependent reflex modulation. Perturbation, load, and obstacle-avoidance studies reveal flexible, goal-dependent corrections inconsistent with passive convergence to fixed referent states. Neural data show continuous preparatory and online control of kinematics and dynamics, contradicting EPH’s central premise. We conclude that, despite its historical influence and contributions, EPH lacks the mechanistic adequacy required of a contemporary theory of motor control and should be succeeded by biologically grounded frameworks that integrate neural processes, biomechanics, and task-level function across basic and applied domains.

## Introduction: the allure of simplicity

Few theories in the history of motor control have attracted as much sustained attention as the equilibrium point hypothesis (EPH). First introduced in the 1960s (Feldman 1966a, b) and repeatedly modified across subsequent decades (Feldman 1986, 2016, 2019; Feldman et al. 1998; Feldman and Latash 2005; Feldman and Levin 2009; Ostry and Feldman 2003), EPH posits that the central nervous system (CNS) specifies equilibrium positions for the limb, leaving peripheral mechanics to execute movement. The hypothesis has been praised for its apparent parsimony and has generated a considerable body of empirical and theoretical work. Importantly, EPH is not merely of historical interest. Its contemporary formulations as “referent configuration/control” continue to be promoted and applied in the 2020s (Feldman and Zhang 2020; Latash 2021, 2022, 2023). Because EPH has shaped experimental paradigms and theoretical interpretations for more than half a century, and most importantly, because it continues to influence current research and clinical applications, it is essential to scrutinize its validity carefully. We are not motivated by a desire to engage in historical nitpicking, but rather by the fact that continuing to rely on EPH carries the risk of perpetuating conceptual difficulties that generate confusion in the field and prevent viable alternatives that are biologically founded from being considered.

Despite our critique, it is important to acknowledge what EPH contributed. First, it offered a unifying lens on posture and movement—framing both as convergence toward referent states—which helped organize experiments and sharpen questions about impedance and stability. Second, it provided a practical intuition for impedance control and the role of muscle–reflex pathways, seeding decades of tractable perturbation paradigms and system-identification work. Third, by foregrounding peripheral mechanics, it made several hard problems experimentally accessible and inspired useful surrogate models—especially for quasi-static or single-joint settings under limited perturbations. These contributions endure. EPH is not a historical artifact but remains an active research program, with contemporary formulations continuing to be developed and applied by Feldman, Latash, and colleagues (Feldman et al. 2015; Feldman 2016, 2019; Latash 2021, 2022, 2023). It is precisely because EPH continues to influence motor control research and clinical practice that systematic evaluation of its adequacy as a comprehensive theory is important. Our claim is not that EPH is fruitless, but that it does not, by itself, account for the physiological richness of human movement. Real coordination demands more than equilibrium specifications: muscles operate under nonlinear force–length and force–velocity laws, reflex pathways are modulated with task-specific precision, and spinal and cortical circuits generate anticipatory and adaptive activity long before equilibrium is ever reached. The hypothesis therefore captures only a slice of motor behavior—quasi-static, single-joint conditions under limited perturbations—while it does not scale to the flexible, whole-body, temporally precise, and goal-dependent coordination observed under realistic conditions.

The central claim of EPH is that motor control can be simplified by exploiting the interaction between musculoskeletal mechanics and reflex pathways, particularly those involving the muscle spindle and associated short- and long-loop circuits. In this formulation, descending commands specify a motor neuron threshold—denoted $\lambda$—that defines the length at which a muscle begins to generate active force. The $\lambda$ parameter is taken to correspond to a referent configuration of the limb, with the realized configuration depending on external forces and boundary conditions. Because force–length properties are assumed to remain stable across the physiological range, specifying $\lambda$ presumably reduces the control problem to setting equilibrium points. This feature is promoted as unifying posture and movement under a common principle: when external loads are absent, the limb is expected to settle passively into a stable posture corresponding to the referent configuration.

The $\lambda$-model formalizes this idea by treating muscle activation as dependent on a threshold muscle length (Feldman 1986; Feldman et al. 1998; Feldman and Levin 2009). In Feldman’s formulation, the threshold length $\lambda ^*$ is defined by descending commands with velocity-, bias-, and noise-dependent terms,

1 $$\begin{aligned} \lambda ^* = \lambda - \mu v + p + \epsilon (t), \end{aligned}$$

and a muscle becomes active only if the current length *l* exceeds this threshold ($l\!-\!\lambda ^*\!>0$). Activation is then proportional to the excess length,

2 $$\begin{aligned} A = [\,l - \lambda ^*\,]^+, \end{aligned}$$

where $[x]^+\!=\!\max (x,0)$ denotes rectification. Muscle force depends on activation, velocity, and time,

3 $$\begin{aligned} F = f(A, v, t). \end{aligned}$$

**Fig. 1:**
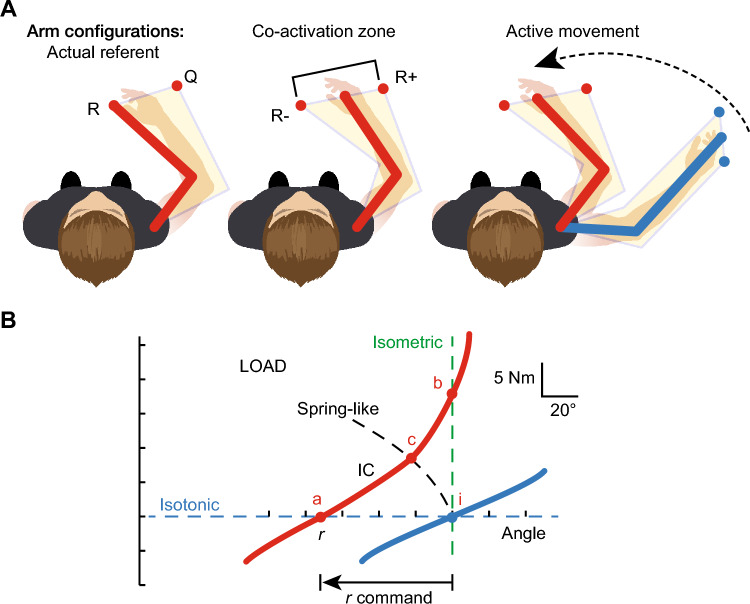
Equilibrium point hypothesis (EPH). **A** (left–middle–right): Planar-arm cartoon viewed from above. Red depicts the *referent* (desired) configuration implied by the descending threshold command; blue depicts the *actual* configuration. Left: Initial state with referent *R* and actual *Q*. Middle: Co-activation zone bounded by lower and upper referent limits ($R_{-}$, $R_{+}$), reflecting stiffness modulation by agonist–antagonist co-activation. Right: Active movement produced by shifting the referent toward a new posture (dashed arrow), which drives the actual limb (blue) to the new equilibrium. **B** Joint-level depiction. The red curve is the *invariant characteristic* (IC) relating joint torque to joint angle for a given threshold command *r* (joint-space analog of $\lambda$); the blue curve is the external load characteristic. Dashed green (vertical) and dashed blue (horizontal) indicate isometric and isotonic constraints, respectively. Intersections (points a–c) are equilibrium states for different *r* or loads; shifting *r* translates the IC and moves the equilibrium without explicitly computing trajectories or forces. Adapted from Feldman (2009)

This framework captures how descending commands shift the threshold of motor-unit recruitment, with muscle force emerging from the interaction of length-, velocity-, and reflex-dependent properties (Fig. 1A, B). At the joint level, the same control appears as a *referent coordinate* *r* (Fig. 1B), which is the joint-space analog of the muscle-space threshold $\lambda$: changing *r* (or $\lambda$) translates the red invariant characteristic (IC) along the angle axis and thereby moves the equilibrium where it intersects the external load (blue).

A common simplification captures the threshold principle in a spring–damper form:

4 $$\begin{aligned} F = k\, (l-\lambda )^+ \;-\; b\,\dot{l}, \end{aligned}$$

where *k* is a stiffness gain and *b* a damping coefficient. Here $l\!-\!\lambda$ corresponds to the joint-space error relative to the referent *r* in Fig. 1B; rectification ensures force develops only when $l\!>\!\lambda$. On the surface, this suggests that specifying $\lambda$ (or equivalently, the central command $\mu$ via *r*) suffices for motor control, with movement dynamics emerging automatically from peripheral mechanics. In practice, however, determining $\lambda$ still requires solving the same inverse problems faced by any theory of movement—mapping desired outcomes onto neural commands, adapting to changing biomechanics, coordinating redundant degrees of freedom, and maintaining stability under perturbation. Thus, complexity is not eliminated but displaced into the choice of the threshold parameter that an unspecified neural mechanism must set.

At its most general level, then, EPH reduces movement generation to shifts in equilibrium configurations defined by $\lambda$ thresholds. Within this account, the CNS is largely exempted from representing forces, torques, trajectories, obstacles, or timing constraints, with such complexities presumed to emerge from the interaction between equilibrium points and external mechanics. The resulting picture is one of extreme simplification: movement appears to arise as a byproduct of mechanical equilibration, rather than as the outcome of context-sensitive neural computation (Bizzi et al. 1992; Feldman 1986, 2016; Feldman et al. 1998; Feldman and Levin 2009; Feldman and Zhang 2020; Latash 2021, 2022, 2023; Ostry and Feldman 2003).

The elegance of this proposal has made it attractive in a domain characterized by complexity, however, several empirical and theoretical limitations have also been presented. The literature has suggested that EPH misrepresents fundamental muscle mechanics (Gomi and Kawato 1996; Gottlieb 1998), disregards critical evidence on neural control (Hinder and Milner 2003; Sainburg 2015), and fails to account for observed motor behaviors (Bizzi et al. 1992). Most importantly, the framework implicitly relies on a homunculus: an unspecified neural mechanism that must calculate the very referent configurations that the hypothesis treats as given (Kenny 2016; Nobre 2001; see also Loeb 2010, 2012). Therefore, it has been suggested that EPH does not resolve the problem of motor control but rather displaces it, by relocating the control problem to an undefined locus.

In this critique, we provide a systematic reassessment of EPH by integrating perspectives from biomechanics, neurophysiology, computational modeling, and other scientific domains. We argue that the enduring appeal of EPH lies less in its explanatory adequacy than in its intuitive simplicity, disciplinary inertia, and the tendency to equate formalized equations with theoretical depth. At the core of the hypothesis remains the unacknowledged assumption of a hidden controller responsible for computing equilibrium points—a circular premise that undermines its scientific credibility.

## Historical context

### The intellectual climate of oversimplification

To understand how the equilibrium point hypothesis (EPH) gained disproportionate prominence, it is necessary to consider the intellectual climate in which it arose. The 1950s through the 1970s were marked by optimism that complex biological phenomena could be reduced to simple mechanical principles. This reductionist enthusiasm drew heavily on the cybernetic movement’s promise to unify biological and mechanical systems under common principles of control and communication (Wiener 1948), and proved particularly influential in motor control.

EPH emerged in this context as a form of what we call “mechanical substitutionism”—the belief that biological systems could evade the computational demands of motor control through leveraged mechanical properties. The equilibrium point framework embodied this tendency, proposing that the CNS need only specify equilibrium positions, with passive muscle and reflex properties accounting for execution (Feldman 1986; Feldman et al. 1998; Feldman and Levin 2009).

The attraction of such an approach is understandable. The motor control problem, as articulated by Bernstein and others, is formidable: the nervous system must coordinate hundreds of muscles across dozens of degrees of freedom, integrate diverse sensory inputs, adapt to unpredictable environments, and achieve precise behavioral goals—all under severe constraints of time, energy, and computational resources (Bernstein 1967; Loeb 2012; Turvey 2007). Therefore, a theory that appeared to dissolve these challenges through mechanical simplicity was naturally compelling.

Yet this appeal rested on a fundamental concept: the assumption that mechanical regularities could substitute for neural computation. As discussed below, the EPH framework does not eliminate the computational problem of motor control. It merely displaces it to an implicit homunculus, which must possess the very computational capacities that EPH was meant to avoid. Recognizing however this historical context is important because it clarifies why EPH has persisted over the years.

### The theoretical appeal: why EPH achieved prominence

To understand EPH’s ability to achieve prominence, it is necessary to examine the specific theoretical advantages that made the framework compelling to motor control researchers. The hypothesis addressed several longstanding problems that we present below.

#### Postural-movement unification

EPH’s primary theoretical contribution was the unification of postural control and voluntary movement under a single mechanistic framework. Traditional approaches treated these phenomena as distinct: postural control involved maintaining equilibrium against external perturbations, while movement control required generating forces to achieve new configurations (Massion 1994; Winter 1995). EPH collapsed this distinction by proposing that both reflected convergence toward nervous system-specified equilibrium positions. Posture represented static convergence at fixed reference configurations, while movement emerged from shifts between equilibrium states.

This unification was supported by empirical observations that limb stiffness varies systematically across postural and movement contexts (Hogan 1985a; Mussa-Ivaldi et al. 1985). The arm’s endpoint stiffness during reaching exhibits directional anisotropy and magnitude modulation that correlate with task demands, creating the appearance of active equilibrium point specification. Similarly, postural responses to perturbations show spring-like restoring forces proportional to displacement magnitude, consistent with equilibrium-seeking behavior (Winter 1995).

#### Dimensional reduction and Bernstein’s problem

EPH appeared to resolve Bernstein’s (1967) degrees of freedom problem through dimensional compression. Rather than coordinating hundreds of muscles individually, the nervous system need only specify equilibrium configurations for limb segments, with peripheral mechanics constraining muscle activation patterns. This formulation suggested that complex coordination could emerge from simple parametric control, bypassing the combinatorial explosion inherent in direct muscle-by-muscle specification.

#### Physiological grounding

Unlike purely computational theories, EPH grounded its predictions in specific neural mechanisms, particularly the muscle spindle-$\gamma$ motor neuron system (Hasan and Karst 1989; Prochazka 1989; Stein and Capaday 1988). The $\lambda$ parameter was identified with $\alpha$-motor neuron activation thresholds, modulated by $\gamma$-motor neuron activity and shaped by descending commands from higher brain centers. This mechanistic specificity distinguished EPH from abstract control theories and provided testable physiological predictions.

The framework also aligned with broader principles of embodied control, where peripheral “smart” mechanisms reduce central computational demands (Pfeifer and Bongard 2006). EPH exemplified this approach by demonstrating how muscle-reflex properties could contribute to behavioral control, relieving the central nervous system of detailed trajectory planning and force computation.

#### Methodological tractability

EPH generated experimentally accessible research paradigms using standard biomechanical techniques. The hypothesis made specific predictions about limb impedance, equilibrium positions, and perturbation responses that could be measured with available technology (Bizzi et al. 1992; Flash 1987; Mussa-Ivaldi et al. 1985). This methodological accessibility enabled extensive empirical investigation and created research programs that sustained theoretical development over decades.

#### Mathematical formalization

The $\lambda$-model added a level of quantitative precision that set EPH apart from earlier accounts of motor control. By introducing equations for threshold modulation, equilibrium position specification, and mechanical convergence, it presented an appearance of rigor comparable to established frameworks in physics and engineering (Feldman 1986; Feldman et al. 1998; Feldman and Levin 2009). This mathematical formalism not only supported computational modeling but also provided a basis for systematic, quantitative comparisons with empirical data, further enhancing the model’s appeal.

#### Clinical applicability

Beyond its theoretical contributions, EPH has influenced clinical motor control research and rehabilitation practice. The framework has provided conceptual foundations for assessing motor impairments following stroke and other neurological injuries, particularly in understanding abnormal muscle tone, spasticity, and impaired reflex modulation (Levin 2000; Musampa et al. 2007; Subramanian et al. 2018). EPH-inspired approaches have shaped therapeutic interventions targeting normalization of reflex thresholds and improvement of functional movement patterns through task-specific training (Feldman and Levin 2009). The framework’s emphasis on threshold position control and reflex modulation has proven useful for developing rehabilitation protocols and understanding recovery mechanisms (Calota and Levin 2009), even when its broader theoretical claims remain contentious.

These apparent advantages explain EPH’s initial acceptance. However, by focusing attention on peripheral mechanics and mathematical formalism, EPH’s proponents and critics alike overlooked a more basic question: what mechanism computes the equilibrium points that the theory treats as given? This oversight reflects a conceptual issue with EPH—the implicit assumption of a computational homunculus.

## The computational homunculus: EPH’s central limitation

### The homunculus problem in motor control

The *homunculus problem* refers to a persistent misconception in neuroscience and cognitive science: explaining a phenomenon by invoking an internal agent that already possesses the very capacities under investigation (Kenny 2016; Nobre 2001). In its simplest form, it entails assuming the existence of a “little man in the head”—a hypothetical inner controller that perceives, decides, and acts on behalf of the organism. Such explanations are circular, as they defer rather than resolve the explanatory burden: the homunculus would itself require the same perceptual, cognitive, and motor abilities that the theory set out to explain.

**Fig. 2:**
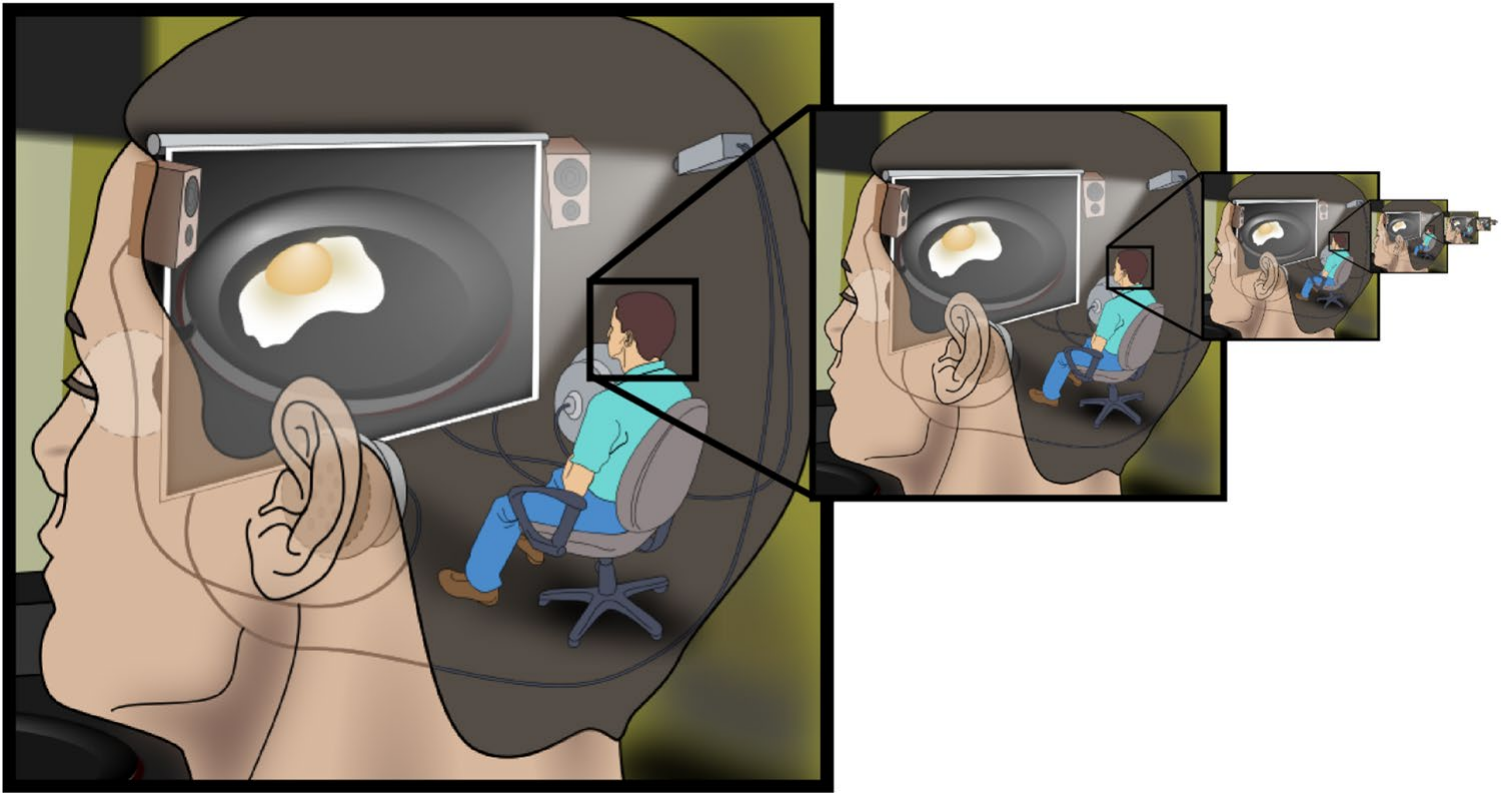
The computational homunculus problem in motor control theories. Many motor control frameworks, including the equilibrium point hypothesis, implicitly assume an internal controller capable of solving the very problems they claim to bypass. EPH posits that the CNS specifies equilibrium points ($\lambda$) to control movement but does not explain how these values are “computed.” Who determines appropriate $\lambda$ values for reaching around obstacles? Who adapts them to novel force fields? Who coordinates them across hundreds of muscles? The framework displaces rather than resolves the motor control problem, invoking an unspecified neural mechanism (the homunculus) that must possess the computational sophistication EPH was designed to eliminate. This creates an infinite regress: the homunculus itself would require explanation, leading to a homunculus within a homunculus. The figure illustrates this circular logic: explaining control by invoking a controller that itself requires control. This fundamental conceptual flaw undermines EPH’s claim to simplify motor control and applies broadly to theories that posit internal models, planners, or optimizers without mechanistic grounding (Image credit: Jennifer Garcia, CC BY-SA 2.5)

In motor control, this problem arises when models explain coordination by presupposing internal mechanisms capable of solving the very control problems under investigation. For example, theories that invoke an internal “planner” that computes optimal trajectories, or an internal “controller” that specifies detailed muscle forces (e.g., Ito 2008; Kawato 1999; Schaal and Schweighofer 2005; Shadmehr and Krakauer 2008; Wolpert et al. 1998; Wolpert and Ghahramani 2000, implicitly reintroduce the homunculus (Fig. 2). These accounts displace the challenge to a hidden agent rather than offering a mechanistic account of how movement emerges from distributed neural, biomechanical, and environmental processes (Mangalam 2025a, c).

With respect to EPH, the hypothesis portrays motor control as simplified by shifting computational responsibilities to peripheral mechanics. It presupposes a mechanism capable of specifying equilibrium points across diverse behavioral contexts. Such an implicit computational homunculus must already possess the very capacities—solving inverse problems, adapting to context, coordinating redundancy—that EPH was meant to bypass, thereby undermining its claim of genuine simplification.

A simple thought experiment illustrates the issue. Consider reaching to grasp a coffee cup while avoiding obstacles on a table. According to EPH, this movement can be produced by specifying appropriate equilibrium points for the limb segments. However, generating these specifications would require a mechanism capable of:

- Processing visual information about target location, obstacles, and limb configuration
- Solving the inverse kinematics problem to identify feasible joint angle configurations
- Planning obstacle avoidance strategies and modifying equilibrium points accordingly
- Integrating proprioceptive feedback to monitor movement progress
- Adapting equilibrium point specifications based on changing task conditions

Each of these requirements constitutes a major challenge in motor control research (Bizzi et al. 1992; Gomi and Kawato 1996; Gottlieb 1998; Hinder and Milner 2003; Sainburg 2015). EPH does not provide solutions to these problems but instead assumes their resolution, thereby attributing sophisticated behavior to an equally sophisticated but uncharacterized internal agent.

### The lambda specification problem

The $\lambda$ specification problem represents the most concrete manifestation of this dilemma. In EPH, motor neuron threshold $\lambda$ is specified through descending commands and corresponds to a muscle length under given mechanical conditions. Yet the theory does not provide a mechanism for how these values are determined. This omission is not a minor technical gap, but a core conceptual issue: without a principled means of specifying $\lambda$, the framework is circular, presupposing precisely the computations it claims to eliminate.

The computational requirements of $\lambda$ specification are considerable, even for simple actions. To execute a reach, the system must: (1) transform a desired endpoint into feasible joint configurations, solving inverse kinematics under redundancy; (2) generate a temporal sequence of $\lambda$ values that yield smooth trajectories, effectively solving inverse dynamics while accounting for inertia, torques, and gravity; (3) adapt $\lambda$ specifications to environmental conditions such as obstacles, loads, or surfaces; (4) coordinate values across many muscles to produce stable synergies, which scale in complexity with muscle number; and (5) monitor execution in real time, adjusting $\lambda$ values with sensory feedback. Each of these problems requires mechanisms of considerable sophistication, none of which are specified by EPH.

Proponents have argued that the appeal to multi-muscle synergies (Latash 1992, 2010; Ambike et al. 2016; Latash et al. 2008) mitigates this complexity. However, the move from single-muscle to group-based $\lambda$ specification simply redistributes the problem. The system must still determine synergy membership, covariation rules, and context-dependent reorganization. Rather than reducing computational complexity, this approach expands it, as the system must coordinate both individual $\lambda$ values and their group-level couplings. Without an explicit mechanism for such processes, EPH shifts the homunculus from single muscles to muscle groups without eliminating it.

### The temporal coordination problem

A further difficulty arises in explaining temporal coordination. Human movements exhibit precise and context-dependent timing relationships across kinematic, kinetic, and neural levels—linking joint rotations and limb trajectories (Stanczyk et al. 2023; Thomas and Gibson 2007), the forces and torques that generate them (Garcia-Retortillo and Ivanov 2022; Hawkes et al. 2019; Ivanenko et al. 2005; Klein Breteler et al. 2007), and neural events orchestrating their execution (Diedrichsen et al. 2007; Sober et al. 2018; Van Acker et al. 2016). These timing relationships are flexible, adapting to task demands, environmental conditions, and biomechanical variability (Lorås et al. 2013; Malone et al. 2012; Pehlevan et al. 2018; Wang et al. 2018).

EPH proposes that temporal coordination emerges from the mechanical properties of the neuromuscular system as equilibrium points shift. However, this view encounters an unresolved specification problem: how are the time courses of equilibrium point shifts generated? To account for human data, any mechanism would need to support:

- *Phase relationships* Different joints initiate and terminate their contributions with specific timing relations that vary with distance, direction, and speed (Galloway and Koshland 2002; Lacquaniti and Soechting 1982).
- *Velocity profiles* Human movements exhibit characteristic acceleration–deceleration patterns that cannot be explained by passive convergence to equilibrium states (Flash and Hogan 1985; Gomi and Kawato 1996; Nagasaki 1989).
- *Multi-phase actions* Everyday movements (e.g., picking up a pencil) require temporally distinct coordination patterns across reach, grasp, and lift phases (Armbrüster and Spijkers 2006; Mangalam et al. 2021).
- *Adaptive timing* Movement timing adapts nonlinearly to changes in task constraints, such as movement distance, accuracy demands, and external loads (Bertucco et al. 2013; Danion et al. 1999; Raket et al. 2016).

Each of these requirements implies advanced mechanisms for generating and adjusting temporal patterns. Without such mechanisms, EPH invokes yet another homunculus—a system implicitly endowed with the very computational abilities that the theory seeks to avoid.

## Theoretical foundations and their flaws

The appeal of the equilibrium point hypothesis has always rested on its promise of theoretical elegance. By reducing the complexity of motor control to equilibrium specifications, EPH appears to offer a unifying framework grounded in simple mathematical formulations and peripheral mechanics. Yet when examined in detail, these foundations reveal several limitations. Below, we consider three major domains of concern. First, the $\lambda$-model itself introduces a mathematical formalism that misrepresents core properties of muscle physiology. Second, the assumption that movements can be governed through equilibrium points alone breaks down under realistic mechanical conditions. Third, the broader strategy of simplifying motor control by displacing computation to the periphery creates new problems rather than resolving old ones.

### The lambda model: mathematical formalism and biological content

#### The threshold activation fallacy

The $\lambda$-model treats muscle activation as binary—silent until muscle length exceeds $\lambda$, after which activation rises linearly. However, electromyographic evidence has demonstrated that motor units are recruited gradually, following Henneman and Olson ’s (1965) size principle, producing smooth, graded force output long before any mechanical threshold is crossed (Jones et al. 2002; Slifkin and Newell 2000). Activation also depends on velocity demands, anticipated loads, co-contraction for stability, and feedforward adjustments to interaction torques (De Luca et al. 1982; Enoka and Duchateau 2017). This information is all missing from the lambda model.

#### The force–length and force–velocity integration problem

While EPH developers acknowledge force–length and force–velocity relationships (Feldman 1986; Latash 2021), the $\lambda$-model’s mathematical formalism does not explicitly incorporate their nonlinear features into threshold control. The framework includes passive force–length properties and velocity-dependent threshold modulation (the $\mu$ parameter), consistent with its axiom that the CNS does not actively set muscle forces. However, this creates a functional integration problem: nonlinear active force–length properties—including distinct ascending limb, plateau, and descending limb regions determined by sarcomere dynamics (Campbell and Moss 2002; Herzog and Leonard 2002; Gordon et al. 1966; Rack and Westbury 1969)—fundamentally shape movement execution, adaptation to perturbations, and force production capabilities. At low activation, force–length curves are relatively flat; at high activation, they sharpen. These activation-dependent changes in muscle mechanics are not captured by threshold-based control.

Similarly, force–velocity relationships govern concentric force reduction and eccentric force enhancement during dynamic movements (Hill 1938; Komi 2000). The claim that the exact form of the F(L) curve does not matter (Latash 2021) overlooks how these nonlinearities constrain achievable equilibrium states and determine whether specified $\lambda$ values can actually produce intended movements under realistic biomechanical constraints. By treating muscles as elements with fixed mechanical properties modulated only by threshold shifts, the $\lambda$-model discards the activation-dependent and velocity-dependent variability that makes muscle adaptive.

#### The force–velocity decisive limitation

The lambda model does not account for force–velocity relationships in the muscle. Real muscles exhibit complex force-velocity characteristics, with concentric contractions producing less force at higher velocities, isometric contractions producing intermediate force levels, and eccentric contractions capable of producing forces greater than isometric maximum (Edman 1988; Hill 1938; Lieber and Fridén 2000; Zajac 1989).

These force–velocity properties are not merely quantitative details but fundamental characteristics that determine muscle function during dynamic movements (Rodríguez-Rosell et al. 2018; Wilkie 1949). The ability of muscles to produce high forces during eccentric contractions is critical for controlling deceleration phases of movement and for absorbing impact forces (Komi 2000). The reduction in force capacity during rapid concentric contractions limits the forces available for acceleration with consequences for the speed–accuracy trade-offs in human movement (Fitts 1954).

By treating muscles as spring-like elements that overlook threshold recruitment dynamics, nonlinear force–length properties, and force–velocity laws, the $\lambda$-model does not simplify motor control but eliminates fundamental biomechanical realities that make motor control possible.

### Mechanical limits of equilibrium point control

The fundamental premise of EPH is that movements can be controlled through the specification of equilibrium points. This premise rests on mechanical assumptions that are limited when applied to real biological systems. In particular, the theory treats the neuromuscular system as a collection of springs and dampers whose equilibrium positions can be independently specified, overlooking the complex mechanical interactions that actually govern limb dynamics (Bizzi et al. 1992; Gomi and Kawato 1996; Gottlieb 1998).

#### The multi-joint coupling problem

Modern EPH formulations extend to multi-joint systems via referent configuration control (Feldman et al. 2015; Latash 2010, 2021), which assigns referent states across multiple degrees of freedom. Yet this expansion does not solve the computational specification problem—it enlarges it. In real multi-joint systems, the equilibrium position of any joint depends critically on the mechanical properties and activation states of muscles crossing other joints in the kinematic chain. This coupling means that equilibrium positions cannot be independently specified for individual joints, as assumed by simpler EPH formulations. For example, in a simple two-joint arm, the equilibrium position of the shoulder is influenced not only by shoulder muscles but also by bi-articular muscles that span both shoulder and elbow. Altering elbow muscle activation will therefore shift the shoulder’s equilibrium position, even if shoulder muscle activation remains constant (Gomi and Kawato 1996; Hogan 1985a; Mussa-Ivaldi et al. 1985; Zajac 1989). In realistic multi-joint systems with dozens of muscles, many of which are bi- or multi-articular, this coupling creates an exponentially complex set of constraints. Specifying equilibrium positions in such systems requires solving highly coupled, nonlinear equations—precisely the type of computation that EPH purports to bypass.

#### The external force field problem

EPH further assumes that equilibrium positions can be specified independently of external force fields, with the system adapting passively through intrinsic muscle mechanics. This assumption misrepresents the physics of mechanical equilibrium, which depends on the net sum of all internal and external forces, including gravity, inertia, interaction torques between limb segments, and environmental contact forces (Flash and Hogan 1985; Hogan 1985a; Shadmehr and Mussa-Ivaldi 1994; Thoroughman and Shadmehr 2000). During dynamic movements, these forces vary continuously, and equilibrium points specified at movement onset rapidly become inappropriate as dynamics evolve. To maintain task performance, the nervous system must therefore respecify equilibrium points online, effectively reintroducing the very computations EPH was designed to eliminate.

#### The stability problem

Even if equilibrium points could be specified, EPH faces the additional challenge of ensuring stable behavior around those points. Real neuromuscular systems exhibit complex, context- and task-dependent stability properties that depend on muscle activation levels, reflex gain settings, and impedance modulation (Hasan and Karst 1989; Prochazka 1989; Stein and Capaday 1988). The $\lambda$-model assumes that stability emerges automatically from the spring-like properties of muscles, yet decades of research demonstrate that reflex gains are actively and systematically tuned: typically high for maintaining postural stability (Colebatch et al. 2016; Deligiannis et al. 2024; Nashner 1976; Schieppati et al. 1990; Woollacott and Nashner 1982), but reduced for allowing movement flexibility. These adaptive gain modulations are not captured by the fixed mechanics assumed in EPH. Furthermore, different classes of tasks impose incompatible stability requirements: postural tasks demand high impedance to reject disturbances, whereas dynamic movements require lower impedance to preserve efficiency and fluidity (Burdet et al. 2001; Hogan 1985a; Mussa-Ivaldi et al. 1985). Because the $\lambda$-model lacks mechanisms to reconcile these demands, it yields predictions that either underestimate stability during posture or overestimate stiffness during movement, producing models of motor behavior inconsistent with empirical observations.

#### The peripheral limitation problem

EPH assumes that peripheral mechanics can solve problems that are fundamentally informational in nature. Yet mechanical systems—no matter how cleverly engineered—cannot generate the temporal organization, contextual flexibility, or adaptive foresight that human movement requires. Human actions exhibit exquisitely controlled phase and timing relationships between movement components, which flexibly reorganize with changing task constraints (Bertucco et al. 2013; Galloway and Koshland 2002; Lacquaniti and Soechting 1982; Danion et al. 1999; Raket et al. 2016). Crucially, this organization is not reducible to the passive convergence of mechanical elements, but arises from distributed neural, biomechanical, and environmental interactions that actively constrain one another across timescales. The limitation is clearest in anticipatory control: humans routinely shape their movements in advance of perturbations, with adjustments tuned by prior experience, practice, and ecological context (Castiello et al. 1998; Furmanek et al. 2025; Hwang et al. 2009; Kelty-Stephen et al. 2023; Ravi et al. 2021). Such anticipatory adjustments reflect an embodied system that is continuously coupling perception, action, and environment, not a set of springs settling to equilibrium. Peripheral mechanics can dissipate forces, but they cannot account for the proactive, flexible, and task-specific coordination that defines skilled human movement.

Taken together, the problems of multi-joint coupling, external force dependence, stability, and peripheral limitation reveal that equilibrium point control is mechanically impossible in biological systems. Far from simplifying the motor control problem, EPH displaces it into a framework that overlooks fundamental mechanical realities.

### The problem of central simplification

The primary appeal of EPH lies in its promise to simplify the motor control problem by relegating complex computations to peripheral mechanics. This approach, which we term the “central simplification problem,” rests on the assumption that reducing central computational burden necessarily results in a more efficient or biologically plausible control system. However, as demonstrated throughout “The computational homunculus: EPH’s central limitation” and “Mechanical limits of equilibrium point control” sections, EPH does not eliminate computational complexity but rather displaces it to an unspecified mechanism responsible for computing equilibrium points. The specification of appropriate $\lambda$ values across diverse behavioral contexts remains equivalent to solving the inverse kinematics, inverse dynamics, and impedance regulation problems that EPH was designed to avoid. Rather than simplifying motor control, this displacement obscures the underlying computational challenges by embedding them within parameters whose determination the framework does not address.

## Neurophysiological evidence

### Spinal circuit organization: pattern generation without equilibrium seeking

The organization of spinal circuits provides strong evidence against the assumptions of the EPH regarding peripheral control. Rather than functioning as simple mechanical relays that implement equilibrium-seeking behavior via stretch reflexes, spinal interneuronal networks exhibit distributed pattern-forming capabilities that shape movement dynamics independent of explicit equilibrium point specifications.

#### Intrinsic spinal pattern formation

Classical experiments demonstrated that rhythmic stepping persists in spinalized and decerebrate animals even when both descending and peripheral inputs are eliminated (Brown 1914; Grillner 1975). This led to the central pattern generator (CPG) concept, according to which spinal interneuron circuits autonomously produce organized locomotor rhythms. Subsequent research has elaborated these models into half-center oscillators, unit burst generators, and multi-level architectures that separate rhythm generation from pattern formation (McCrea and Rybak 2008; Guertin 2009). These circuits generate systematic amplitude, phase, and frequency relationships through intrinsic connectivity and neuromodulatory influences (Guertin 2009). Such autonomous temporal structuring directly contradicts the EPH assumption that movement timing emerges passively from mechanical convergence toward equilibrium states.

#### Flexible adaptation

Crucially, spinal pattern generators are not rigid oscillators but reorganize dynamically in response to sensory input, descending drive, and neuromodulatory state, producing context-dependent variations in locomotor output (McCrea and Rybak 2008; Guertin 2009). Contemporary computational studies further show that robust rhythmic coordination can emerge even from sparse or irregular neuronal firing patterns, reflecting population-level balance between excitation and inhibition (Strohmer et al. 2024). Similarly, recent work highlights that spinal CPG networks in mammals retain the capacity to adapt after injury, reorganizing gait patterns autonomously in the absence of supraspinal input (Su et al. 2025). These findings underscore that spinal circuits actively generate, structure, and adapt motor patterns, a capability fundamentally incompatible with equilibrium-based formulations that lack intrinsic pattern-forming and adaptive mechanisms.

#### Propriospinal networks and multi-segmental coordination

Propriospinal interneurons (PNs) provide an anatomical and functional substrate for coordinating activity across multiple spinal segments. Early work demonstrated that long descending and ascending propriospinal pathways allow reflex irradiation and interlimb coordination, challenging the notion that spinal control is strictly local or segmental (Sherrington and Laslett 1903). Quantitative anatomical studies have since shown that a substantial fraction of fibers in the ventral and lateral funiculi are propriospinal, with both myelinated and unmyelinated components contributing to intra-spinal communication (Chung and Coggeshall 1983). Modern circuit-tracing approaches have confirmed that long premotor propriospinal neurons form direct connections between cervical, thoracic, and lumbar levels, linking forelimb, trunk, and hindlimb motor pools (Ni et al. 2014). These neurons propagate locomotor commands rostrocaudally and contribute to left–right alternation and flexor–extensor coordination, indicating that propriospinal relays are integral components of central pattern-generating circuits rather than passive conduits (Laliberte et al. 2019).

Their connectivity is highly dynamic. Descending drive, sensory input, and neuromodulation reconfigure propriospinal coupling patterns to support task-specific demands such as gait speed, load compensation, and interlimb coordination (Laliberte et al. 2019). Recent work has revealed extensive plasticity in these networks: following spinal cord injury, propriospinal interneurons sprout new projections and form detour circuits that bypass lesions, restoring partial locomotor output in both animal models and humans (Cheng and Guan 2023; Dominguez-Bajo and Clotman 2024). This capacity for structural remodeling and adaptive coordination contradicts EPH’s assumption that spinal control is governed by fixed mechanical properties.

Propriospinal networks also integrate multimodal sensory information, comparing afferent inputs with ongoing motor states and generating context-dependent motor responses (Kostyuk and Vasilenko 1979; Laliberte et al. 2019). Such flexible computation cannot be reduced to monosynaptic reflex arcs and demonstrates that the spinal cord actively contributes to adaptive motor control through propriospinal integration and reconfiguration. These findings reinforce that spinal circuits operate as distributed, plastic control networks, inconsistent with the equilibrium point view of movement as convergence toward mechanically determined equilibria.

#### Reflex modulation and context-dependent control

The systematic modulation of spinal reflexes during voluntary movement provides strong evidence against equilibrium-based accounts. Rather than being fixed mechanical reactions, reflex gains are precisely tuned according to task requirements, movement phase, and strategic goals (Prochazka 1989). For example, stretch reflex amplitude is enhanced when it supports postural stability but suppressed when it would destabilize balance, demonstrating context-specific control rather than passive convergence (Stein and Capaday 1988). The temporal precision of reflex modulation reflects predictive and adaptive computation. Reflex transmission can be altered within tens of milliseconds to accommodate unpredictable perturbations, with phase-dependent modulation of group I and group Ib afferent inputs observed during locomotion (Gossard et al. 1994; Pearson 1995). Reflex pathways also show long-term plasticity, adapting with training and pharmacological state; classic studies demonstrated that interneurons mediating flexor reflex afferent effects reorganize after dopaminergic modulation, shifting their excitatory/inhibitory balance in ways dependent on network state (Jankowska et al. 1967). Such findings highlight that reflex control is embedded in dynamic circuit-level organization, not fixed mechanical thresholds.

Contemporary neuromodulation approaches further demonstrate that spinal reflex pathways can be precisely adjusted for functional recovery. Epidural spinal cord stimulation, for instance, engages proprioceptive afferents and propriospinal circuits to enhance or suppress reflex excitability in a spatially and temporally selective manner, promoting adaptive reorganization after injury (Zhang et al. 2025). Together, this body of evidence shows that spinal circuits operate as actively controlled, plastic networks capable of predictive, context-dependent regulation—capabilities that cannot be reconciled with the static mechanical assumptions of EPH.

### Cortical control: constraints on equilibrium point accounts

Neurophysiological evidence accumulated over the past four decades provides compelling evidence against EPH, and these findings continue to expand with advances in multi-neuron recording methods. Direct neural recordings from motor cortex during reaching movements reveal patterns of neural activity fundamentally incompatible with the equilibrium point framework and challenge its core assumptions about neural control mechanisms.

#### Population vector organization and kinematic encoding

Single-unit recordings from primary motor cortex (M1) demonstrate that neural activity is broadly tuned to movement direction, with individual neurons exhibiting preferred directions and contributing across a wide range of directions (Georgopoulos et al. 1982, 1988; Schwartz et al. 1988). This tuning gives rise to a population vector organization, in which ensembles of neurons collectively encode movement vectors—velocity, direction, and trajectory characteristics—rather than equilibrium positions, as posited by the EPH.

The temporal dynamics of cortical activity during reaching further reveal systematic relationships to evolving kinematics across movement execution. Neural firing patterns anticipate and track velocity, acceleration, curvature, and endpoint approach, often with heterogeneous and multiphasic response profiles (Churchland and Shenoy 2007). These time-varying patterns represent precisely the kind of kinematic control that the EPH assumes should emerge passively from peripheral mechanics, rather than through explicit neural representation.

Population-level analyses reinforce this view. Summed activity across M1 neurons, weighted by their preferred directions, produces a population vector that predicts instantaneous velocity and trajectory with high accuracy throughout movement (Georgopoulos et al. 1988; Michaels et al. 2016; Suresh et al. 2020). Continuous encoding of evolving kinematics is therefore inconsistent with equilibrium-based frameworks, which predict only transient specification at movement onset.

#### Preparatory activity and movement planning

Equally challenging for the EPH is the extensive evidence for preparatory neural activity preceding movement. Neurons in premotor and motor cortex exhibit systematic changes during delay periods that correlate with the upcoming direction, amplitude, and speed of intended movements (Churchland et al. 2006; Riehle and Requin 1989; Tanji and Evarts 1976). Such anticipatory activity reflects the specification of movement parameters long before initiation, indicating that the nervous system actively plans forthcoming actions rather than relying on passive convergence to equilibrium states.

The temporal evolution of preparatory activity is structured. Riehle and Requin (1989) showed that information about movement direction modulates preparatory discharge more strongly and earlier than information about extent. Churchland et al. (2006) demonstrated that neural variability in premotor cortex declines progressively during preparation, consistent with the system converging toward an optimal subspace of firing rates predictive of faster reaction times. Kaufman et al. (2014) further revealed that preparatory activity occupies an “output-null” space relative to motor output, allowing detailed specification of future movements while withholding execution until the appropriate go cue.

Recent work has emphasized the flexibility of these preparatory dynamics (Chae et al. 2024). They adapt to task predictability and strategic demands, adjusting the timing and structure of neural trajectories across trials. Preparatory signals are also context-dependent, varying with behavioral goals and environmental constraints. Collectively, this body of evidence demonstrates that preparatory activity embodies complex and flexible neural coordination that cannot be reduced to equilibrium point specification of movement onset.

#### Online control and trajectory modification

Direct cortical recordings during perturbation and obstacle-avoidance tasks demonstrate continuous neural activity throughout movement execution, with responses tightly linked to evolving kinematics and force production (Evarts 1968; Omrani et al. 2017). Perturbations elicit rapid changes in motor cortical discharge within 50–100 ms that are tuned to the direction and magnitude of the disturbance and scale with force output, accurately predicting subsequent corrections (Evarts 1968; Omrani et al. 2017). These latencies are incompatible with recomputing equilibrium points and consistent with rapid feedback control implemented through long-latency pathways that share circuitry with voluntary commands (Pruszynski and Scott 2012).

Perturbation-related responses are directionally tuned and scale with disturbance magnitude, accurately predicting subsequent corrective actions (Omrani et al. 2017). Moreover, cortical responses vary systematically with task demands: instructions emphasizing speed, accuracy, or smoothness alter the magnitude and timing of corrective activity, paralleling behavioral adjustments (Perich et al. 2018). Such task- and context-dependent modulation reveals a level of flexibility and predictive adaptation that is incompatible with the mechanistic, context-independent control postulated by EPH.

## Computational modeling issues

### The simulation gap: models that cannot model

Despite more than five decades of development, computational models based on the EPH have not been able to capture key features of human motor control. Their limitations appear not to stem from computational power, parameter tuning, or simulation methodology, but from deeper theoretical issues in the framework itself. As the following sections illustrate, EPH-based models break down when tested against increasingly realistic conditions: even simple single-joint tasks expose weaknesses, multi-joint simulations fail to reproduce biological coordination, and obstacle-avoidance scenarios highlight incompatibilities with human planning and execution.

#### Simple movement failure under realistic conditions

EPH-based models typically achieve only marginal successes when restricted to highly simplified scenarios involving single-joint movements under idealized conditions with no external perturbations, obstacles, or time constraints. Even these limited successes dissolve rapidly when more realistic conditions are introduced, with a number of unresolved limitations to the EPH debated in the literature for many years, including whether muscle resistance to displacement, measured during movement, is adequate to support this form of control (Bizzi et al. 1992; Gomi and Kawato 1996; Gottlieb 1998; Hinder and Milner 2003; Sainburg 2015).

When external loads are applied to simulated movements, naïve EPH models generate trajectories that deviate grossly from human performance. To overcome these failures, Gribble and Ostry (2000) introduced an iterative adaptation scheme in which control signals are modified in direct proportion to positional error. Although this procedure produces trajectories resembling those of adapted human movements, it does so only by layering on error-driven updates that effectively mimic forward modeling. The apparent success of the model thus depends on increasingly contrived modifications of equilibrium signals rather than any intrinsic explanatory power of the EPH. Even then, the simulations display artifacts such as end-point oscillations, overshoot, and the need for repeated iteration, which underscore the lack of biological plausibility. In practice, the EPH can only reproduce adaptation by introducing additional mechanisms that it was originally designed to avoid.

By contrast, humans systematically adjust to novel loads, reshaping motor commands to preserve accuracy and efficiency (Shadmehr and Mussa-Ivaldi 1994). EPH formulations offer no such flexibility, with their responses being stereotyped consequences of fixed mechanical properties. Genuine adaptation requires dynamic modulation of agonist muscle forces, not post hoc patching of equilibrium signals.

#### Multi-joint coordination issues

When $\lambda$-based accounts are extended from simple single-joint cases to multi-joint systems, their shortcomings are more evident. Reconstructions of putative equilibrium trajectories during multi-joint actions can yield superficially similar timing profiles across joints, yet they fail to reproduce the structured inter-joint coordination observed in humans (Latash 1994). Humans, by contrast, resolve redundancy through flexible synergies that stabilize task-relevant variables while permitting variability in task-irrelevant directions. This pattern is captured by the uncontrolled manifold (UCM) framework, which was originally motivated within the equilibrium point tradition but has since become a broadly applicable method for analyzing coordination (Latash et al. 2001; Scholz and Schöner 1999). Empirically, arm movements show precisely this structured organization: straight or gently curved end-point trajectories, bell-shaped velocity profiles, and systematic inter-joint timing relations that cannot be explained by passive convergence to equilibrium states (Flash and Hogan 1985; Lacquaniti and Soechting 1982).

Multi-joint tests of the equilibrium point hypothesis show that measured joint/endpoint stiffness and long-latency feedback are task- and phase-dependent in ways incompatible with passive convergence to a fixed referent configuration (Gomi and Kawato 1996; Gottlieb 1998). Moreover, when interaction torques or external loads are introduced, models based on shifting equilibria either devolve into erratic, poorly correlated joint trajectories or require rigid, phase-locked scheduling that ignores the efficiency and adaptability evident in human reaching, including compensations for interaction torques and context-specific impedance tuning (Gribble and Ostry 2000; Shadmehr and Mussa-Ivaldi 1994; Thoroughman and Shadmehr 2000). The net result is simulated endpoint behavior that bears little resemblance to the smooth, systematically organized velocity relationships found in biological movement.

Attempts to combine EPH with redundancy-based frameworks have fared no better. The most comprehensive model, developed by Martin et al. (2009), explicitly sought to integrate the uncontrolled manifold hypothesis with equilibrium point control. Yet the simulations still failed to capture human coordination: they generated excessive joint “self-motion,” curved and inefficient end-effector paths, and temporal patterns incompatible with robust, adaptive reaching. Worse still, when perturbations were introduced, instabilities propagated unsystematically through the kinematic chain, yielding movements that would be biomechanically impossible for humans. These issues underscore the inability of EPH formulations to scale beyond highly simplified scenarios—once confronted with the demands of multi-joint coordination, the hypothesis fails to scale under realistic constraints.

#### The obstacle avoidance problem

Perhaps the most decisive demonstration of EPH shortcomings come from obstacle avoidance tasks. Human reaching around obstacles shows systematic, anisotropic path variations reflecting the inertial sensitivity of the arm—trajectories are planned to minimize vulnerability to perturbations when passing closest to the obstacle (Sabes et al. 1998). This sensitivity-based planning yields mirror-symmetric strategies across arms and aligns with the mobility minor axis, indicating that the CNS incorporates biomechanics into motor planning. Such findings are irreconcilable with equilibrium point mechanisms, which cannot integrate obstacle geometry with limb dynamics.

The few attempts to adapt EPH-like frameworks for obstacle avoidance have resorted to introducing artificial repulsive force fields around obstacles (Hogan 1985b; Khatib 1986), effectively abandoning equilibrium point control in favor of potential-field methods. These ad hoc modifications violate the central premise of EPH by requiring explicit conversion of visual information about targets and obstacles into force-level adjustments (Sabes et al. 1998). Even then, the resulting simulated trajectories bear little resemblance to human performance: they display exaggerated deviations, unnatural velocity profiles, and coordination patterns that contradict the efficiency and stability principles evident in empirical obstacle-avoidance movements.

### The parameter problem: unfalsifiable curve fitting

EPH-based models face a fundamental parameter problem that limits their explanatory and predictive power. To simulate even simple behaviors, they require specification of numerous parameters describing muscle mechanics, reflex gains, equilibrium trajectories, and system dynamics. These parameters are not derivable from first principles and must be tuned separately for each task or experimental condition, providing little predictive value outside the fitted context (Goodman and Latash 2006).

#### Parameter proliferation and overfitting

As the framework has been extended, EPH implementations have accumulated increasingly large sets of free parameters. For example, studies of multi-finger force production demonstrated non-monotonic, single-peak variance profiles that could only be reproduced by introducing additional scaling parameters into the models (Goodman et al. 2005). Feed-forward extensions that attempt to reconcile EPH with the uncontrolled manifold hypothesis also require knowledge of Jacobians and other mappings, introducing new assumptions and degrees of freedom that must be estimated empirically (Goodman and Latash 2006). This proliferation reduces the models to descriptive curve fits: with sufficient adjustment of parameters, a wide range of movement patterns can be approximated, but the models provide no principled method for parameter selection or for predicting responses to novel conditions.

#### Physiological implausibility of parameters

A second difficulty is that many EPH parameters lack physiological interpretability. The $\lambda$-parameters central to the framework cannot be directly linked to measurable neural or mechanical quantities (Sainburg 2015). The determination of the so-called invariant characteristic also requires the assumption of a constant central state, a condition that is rarely satisfied in experimental or natural behavior (Sainburg 2015). When parameters are restricted to ranges consistent with known physiological constraints, EPH models generally fail to reproduce empirically observed movements.

#### The learning and adaptation problem

A further limitation of EPH models is their lack of mechanisms for learning and adaptation. In the Feldman–Latash formulation, the controller specifies a fixed equilibrium trajectory or referent configuration, while spinal reflexes are assumed to manage task dynamics and noise (Latash 2010). Within this framework, motor behavior emerges from setting thresholds for muscle activation, and stability is provided by the interaction of these thresholds with musculoskeletal mechanics. Although this approach accounts for certain steady-state features, it excludes principled mechanisms for error-driven modification of control variables.

In contrast, human motor behavior demonstrates systematic adaptation across repeated practice, transfer of learning between tasks, and rapid compensation for environmental changes. For example, Wei et al. (2007) showed that during rhythmic ball-bouncing, humans exploit passive stability but also apply active, proportional error corrections to perturbations, enabling recovery dynamics that are both faster and more systematic than those predicted by purely passive models. These findings suggest that humans flexibly combine passive dynamics with active, experience-dependent adjustments. By comparison, EPH models lack such mechanisms: changes in task conditions can only be accommodated through manual re-specification of parameters by the modeler, rather than through intrinsic processes of adaptation and learning.

This disconnect illustrates that EPH lacks rules for modifying parameters based on performance history, sensory error, or task demands. The absence of such mechanisms means that the framework cannot explain how humans improve with practice or generalize learned skills, reinforcing that EPH remains descriptive rather than predictive.

## What the data actually shows—and what it does not

The EPH rests on a foundation of empirical findings interpreted as supporting its core claims. Below we re-examine three widely cited publications that helped establish and entrench EPH. For each, we first summarize the main empirical or theoretical contribution. We then show why the reported phenomena do not uniquely support EPH and are often more parsimoniously explained by alternative mechanisms when considered alongside broader empirical datasets. Our goal is not to dismiss these contributions or their empirical value, but to clarify what they demonstrate about motor control mechanisms and what remains unresolved regarding EPH’s specific claims. We acknowledge that multiple theoretical frameworks can often account for the same phenomena. Our analysis examines whether these findings provide the unique support for EPH that has been claimed.

### Feldman (1986): “Once more on the equilibrium-point hypothesis”

#### What the paper established

Feldman (1986) formalized the $\lambda$-model, proposing that voluntary posture and movement emerge from shifts in motor neuron activation threshold (the referent configuration). The core empirical claim is the *invariant characteristic:* a presumed stable relation between muscle force and length when the central state is held constant. From this relationship, one can infer a referent length $\lambda$ and, by extension, a joint equilibrium position. The framework suggests that specifying $\lambda$ is sufficient for motor control, with execution emerging from the interaction between this threshold and peripheral mechanics.

#### Why the finding does not uniquely support EPH

While the invariant characteristic can be measured under restricted experimental conditions, several lines of evidence challenge its relevance to natural motor behavior and its necessity for explaining observed phenomena.

First, the invariant characteristic assumes stationarity of the “central state” during measurement. However, reflex gains, co-contraction levels, and fusimotor drive are known to modulate within tens of milliseconds in a task-, phase-, and context-dependent manner (Prochazka 1989; Stein and Capaday 1988). These rapid changes violate the stationarity prerequisite during natural behavior, making the invariant characteristic an experimental artifact of constrained conditions rather than a functional control principle. The framework does not specify how the nervous system determines appropriate $\lambda$ values across continuously changing behavioral contexts.

Second, the $\lambda$-model’s mathematical form treats muscles as thresholded linear springs with force proportional to $(l\!-\!\lambda )^+$. This simplification is incompatible with well-established muscle physiology: muscles exhibit nonlinear force-length curves with distinct ascending limb, plateau, and descending limb regions determined by actin-myosin overlap (Gordon et al. 1966; Zajac 1989); force-velocity relationships show concentric force reduction at higher speeds and eccentric force enhancement (Hill 1938); and history-dependent effects such as residual force enhancement alter force production based on prior contraction history (Edman et al. 1982; Herzog et al. 2006; Joumaa and Herzog 2014). These nonlinearities fundamentally shape movement dynamics and adaptation to perturbations, yet the $\lambda$-model provides no mechanism for integrating them with threshold control or predicting their effects on equilibrium specification.

Third, empirical studies of multi-joint reaching under perturbation reveal that endpoint and joint stiffness are strongly task- and direction-dependent, varying systematically with movement goals, accuracy demands, and anticipated disturbances (Burdet et al. 2001; Gomi and Kawato 1996; Gottlieb 1998). Humans actively modulate impedance geometry—creating anisotropic stiffness fields aligned with task-relevant error dimensions—rather than exhibiting the fixed mechanical properties implied by passive convergence to a referent configuration. This selective impedance regulation requires active neural control mechanisms that EPH does not specify (Burdet et al. 2001; Hogan 1985a; Pruszynski and Scott 2012).

Fourth, long-latency reflex responses ($50\text {--}100$ ms) demonstrate goal-dependent modulation: they can reverse sign with task instruction, align with voluntary goals rather than mechanical disturbance characteristics, and implement near-optimal feedback corrections (Pruszynski and Scott 2012; Yang et al. 2011). These responses blend spinal reflex pathways with cortical influence, exhibiting rapid online control that is inconsistent with fixed threshold mechanics and passive convergence (Perich et al. 2018; Scott 2004). The framework must therefore incorporate supraspinal gain modulation as an auxiliary mechanism, effectively reintroducing the active feedback control it was designed to replace.

#### What remains unexplained

The $\lambda$-model does not address how the nervous system computes appropriate threshold values across varying task conditions, how nonlinear muscle properties integrate with threshold control, or how task-dependent impedance modulation is achieved. The observed spring-like restoring forces during constrained perturbations can be produced by feedback control with appropriate gain settings, without invoking hidden equilibrium trajectories (Hogan 1985a; Pruszynski and Scott 2012).

### Flanagan et al. (1993): “control of trajectory modifications in target-directed reaching”

#### What the paper established

Flanagan et al. (1993) recorded human reaching movements to both fixed and displaced visual targets and compared them with simulations generated by a two-joint arm model based on EPH. They proposed that the equilibrium position (EP) of the hand shifts in a straight line toward the target, with trajectory modifications following target displacement achieved by changing the direction of this EP shift. The study aimed to demonstrate that simple, time-varying control signals shifting equilibrium points could generate the kinematic patterns observed empirically, including straight-line hand paths and bell-shaped velocity profiles.

#### Why the finding does not uniquely support EPH

While the simulations reproduced several features of human reaching, the ability to fit kinematic data with equilibrium-based models does not demonstrate that the nervous system uses equilibrium points as control primitives. Multiple control strategies can produce kinematically similar movements and comparable responses to target displacement, making model fitting fundamentally ambiguous with respect to underlying control mechanisms.

First, the kinematic regularities that Flanagan et al. (1993) successfully simulated—including bell-shaped velocity profiles, roughly straight hand paths, and smooth trajectory modifications—can be generated by optimal feedback control policies that minimize task-relevant costs without positing equilibrium trajectories (Harris and Wolpert 1998; Flash and Hogan 1985). These alternative frameworks produce statistically indistinguishable movement kinematics while implementing fundamentally different computational principles: optimizing expected costs under uncertainty rather than converging toward equilibrium states. The fact that EPH-based simulations can match human kinematics does not rule out these more parsimonious alternatives.

Second, subsequent work on adaptation to novel dynamics reveals control principles incompatible with equilibrium-based accounts. When subjects reach in velocity-dependent force fields (curl fields), they exhibit gradual, trial-by-trial learning over $20\text {--}100$ movements (Shadmehr and Mussa-Ivaldi 1994). This adaptation shows state-dependent force pattern learning, generalization in extrinsic workspace coordinates rather than muscle or joint coordinates (Krakauer et al. 2000), savings upon re-exposure, and interference between opposing force fields (Smith et al. 2006). These characteristics indicate learning of internal dynamics models—mechanisms for predicting how actions produce forces—rather than simple retuning of equilibrium point trajectories or threshold recalibration (Thoroughman and Shadmehr 2000). The gradual nature of adaptation and its workspace generalization structure point to active learning mechanisms that EPH does not provide.

Third, obstacle-avoidance trajectories reveal planning that incorporates limb dynamics and biomechanical constraints in ways incompatible with simple equilibrium-point shifting. Sabes et al. (1998) demonstrated that reaching paths around obstacles exhibit anisotropic deviations aligned with the arm’s mobility ellipse—the direction of greatest inertial sensitivity—indicating that trajectory selection reflects risk assessment based on limb dynamics. This planning cannot be captured by potential-field methods or EPH-based trajectory generation without ad hoc assumptions about how obstacle information integrates with referent configuration specification. The sensitivity-based trajectory selection suggests that the nervous system explicitly represents and exploits biomechanical properties during planning, contradicting EPH’s claim of minimal central representation and peripheral execution.

Fourth, neural population dynamics during reaching provide evidence for continuous, active control rather than brief equilibrium specification. Motor cortical populations exhibit time-varying activity throughout movement preparation and execution (Churchland et al. 2012; Georgopoulos et al. 1988), with neural firing patterns that correlate with instantaneous velocity, force, and trajectory curvature. Following target displacement or mid-movement perturbations, cortical responses occur within $50\text {--}100$ ms and predict subsequent corrective actions (Evarts 1968; Omrani et al. 2017; Perich et al. 2018). This persistent neural engagement throughout movement contradicts the notion of brief equilibrium point specification at movement onset followed by passive mechanical convergence, and instead indicates ongoing active control that continuously shapes trajectory execution.

Fifth, the model’s assumption of simple, time-varying control signals (linear shifts in equilibrium position) glosses over the computational challenge of determining appropriate signal parameters. How does the nervous system compute the rate and direction of equilibrium point shifts? How are these parameters adjusted for different workspace locations, movement speeds, accuracy requirements, and external loads? The framework does not specify the mechanisms that generate these control signals, effectively displacing rather than solving the motor control problem—the very computational homunculus we identified earlier. As noted by Hinder and Milner (2003) and Gottlieb (1998), the requirement to specify appropriate equilibrium trajectories reintroduces the same inverse dynamics and planning problems that EPH was intended to bypass.

#### What remains unexplained

The successful kinematic fitting achieved by Flanagan et al. (1993) demonstrates that equilibrium-point models constitute one possible description of movement data but does not establish them as the neural control strategy. Feedback control with online gain adjustment (Scott 2004), predictive dynamics compensation (Shadmehr and Mussa-Ivaldi 1994), and optimal control under uncertainty (Harris and Wolpert 1998) can produce the same empirical movement patterns—including straight-line paths, bell-shaped velocities, and smooth trajectory corrections—without invoking equilibrium points as control primitives. The critical question is not whether EPH-based models can fit data, but whether they provide the most parsimonious account that integrates motor planning, learning, adaptation, neural dynamics, and biomechanical constraints. On these broader criteria, equilibrium-based accounts face substantial challenges that alternative frameworks—despite their own limitations—address more naturally than EPH.

### Latash (2010): “motor synergies and the equilibrium-point hypothesis”

#### What the paper established

Latash (2010) synthesized EPH with the uncontrolled manifold (UCM) framework, proposing that the CNS specifies referent states for task-level variables while allowing structured variability (motor synergies) along goal-equivalent directions. This influential integration positioned EPH as providing a mechanistic substrate for synergy formation: equilibrium point control naturally produces coordination patterns that stabilize task-relevant variables while permitting flexibility in task-irrelevant dimensions. The framework suggested that synergies emerge from groups of muscles sharing common referent configurations.

#### Why the finding does not uniquely support EPH

While the UCM framework successfully characterizes coordination structure across diverse motor tasks, this structure does not require or uniquely support equilibrium point control. The relationship between UCM patterns and control mechanisms is indirect, with multiple frameworks capable of producing UCM-consistent variability structure.

First, UCM structure—greater variability along goal-equivalent manifolds than along error-producing directions—indicates that the nervous system stabilizes task-relevant variables while allowing flexibility elsewhere (Scholz and Schöner 1999; Latash et al. 2001). However, this pattern emerges naturally from any control strategy that selectively weights task-relevant errors, including optimal feedback control (Scott 2004), impedance control with directional stiffness modulation (Hogan 1985a), and task-space controllers that explicitly regulate goal-relevant coordinates. None of these alternatives requires $\lambda$-specification or equilibrium point primitives; they produce UCM structure through selective stabilization of task spaces via feedback gains, impedance geometry, or cost function shaping.

Second, computational implementations reveal fundamental limitations when EPH is embedded within UCM-based coordination. Martin et al. (2009) developed the most comprehensive attempt to integrate EPH with UCM principles for multi-joint reaching. Despite careful implementation, their simulations produced systematic deviations from human performance: excessive joint “self-motion” (large joint excursions with minimal endpoint displacement), curved rather than straight hand paths, inefficient coordination patterns, and instabilities under perturbation that required non-EPH corrective mechanisms. When external perturbations were applied, the models exhibited cascading instabilities and biomechanically implausible joint configurations. These failures occurred despite extensive parameter tuning and indicate that equilibrium point control does not naturally scale to multi-joint coordination, even when augmented with synergy constraints (Gomi and Kawato 1996; Gottlieb 1998).

Third, practical implementations of EPH–UCM models suffer from severe parameter proliferation. Reproducing even simple reaching movements requires task-specific manual tuning of referent trajectories, stiffness coefficients, and damping parameters (Goodman et al. 2005; Goodman and Latash 2006). Parameters must be re-specified for each task context, movement direction, and perturbation condition, preventing generalization and falsifying claims of theoretical parsimony. When Gribble and Ostry (2000) attempted to model adaptation to force fields using EPH, they required iterative error-correction schemes that effectively implement the feedforward compensation mechanisms EPH was designed to avoid. The augmented models bear little resemblance to the original equilibrium point formulation.

Fourth, synergy formation itself requires mechanisms that EPH does not specify. How are muscles grouped into functional units sharing referent configurations? How do these groupings reorganize with task demands, skill acquisition, and environmental constraints? What principles govern the selection and weighting of synergies? The framework treats synergies as given organizational structures rather than explaining their origin, flexibility, or context-dependent modulation.

#### What remains unexplained

UCM structure demonstrates task-specific coordination but does not reveal the underlying control mechanisms. Feedback control with selective impedance modulation along task-relevant dimensions can reproduce UCM patterns across hands, fingers, and limbs without invoking referent configurations: the controller stiffens error-sensitive directions and relaxes goal-equivalent ones via appropriate gain matrices and impedance geometry (Hogan 1985a; Scott 2004). Constraint-based and neuromechanical accounts further show how synergies can emerge from task-level objectives and plant constraints, reorganize with context, and generalize with practice—providing explicit mechanisms for formation, adaptation, and scaling that equilibrium-point formulations lack (Bizzi and Cheung 2013; Bruton and O’Dwyer 2018; Cheung and Seki 2021; McKay and Ting 2012; O’Reilly and Delis 2024; Razavian et al. 2015; Ting and McKay 2007).

### Synthesis: the pattern of auxiliary hypotheses

Examination of these foundational papers reveals a consistent pattern: empirical observations initially cited as supporting EPH either (1) do not uniquely support the framework when alternative mechanisms are considered, (2) require auxiliary assumptions that undermine EPH’s claimed simplicity and parsimony, or (3) directly contradict core predictions when examined in realistic behavioral contexts.

The invariant characteristic requires stationarity assumptions that are systematically violated during natural behavior; task-dependent reflex modulation requires active supraspinal gain control mechanisms; adaptation to novel dynamics requires learning mechanisms that EPH does not specify; synergy formation and reorganization require organizational principles that the framework lacks; and multi-joint coordination requires solutions to inverse kinematic and dynamic problems that equilibrium point specification was originally meant to bypass. As each empirical challenge emerges, modern EPH formulations incorporate additional mechanisms—continuous $\lambda$ updates, cortical involvement in specification, impedance regulation, error-driven adaptation (Feldman and Levin 2009; Feldman 2011, 2016, 2019; Feldman et al. 2015; Latash 2010)—that progressively transform the framework into a variant of the feedback control theories it claimed to replace, while retaining the terminology of equilibrium points.

Table 1 systematizes this critique across all core EPH assumptions, identifying contradictory evidence and assessing implications for the framework’s viability, parsimony, and falsifiability.

**Table 1:** Systematic evaluation of equilibrium point hypothesis (EPH) claims against empirical evidence

EPH core assumption	Contradictory empirical evidence	Implications for EPH
*Movement as passive convergence:* Shifting $\lambda$ (referent configuration) drives execution via spring-like muscle mechanics; CNS need not represent trajectories or forces	Rapid goal-dependent corrections (< 100 ms), anticipatory EMG preceding predictable perturbations, and continuous cortical activity tracking evolving kinematics all indicate active, ongoing neural control incompatible with one-time equilibrium specification	*Requires auxiliary assumption:* Continuous $\lambda$ respecification throughout movement, which reintroduces the trajectory planning problem EPH claimed to eliminate. *Parsimony cost:* High
*Muscle mechanics as thresholded linear springs:* Force proportional to $(l\!-\!\lambda )^+$ with fixed stiffness coefficient	Muscles exhibit nonlinear force-length curves with ascending limb, plateau, and descending limb; force-velocity relationships show concentric force reduction and eccentric force enhancement; history-dependent residual force enhancement	*Fundamental incompatibility:* The $\lambda$-model’s mathematical form cannot capture established muscle properties. Modern EPH acknowledges nonlinearities exist but provides no mechanism for how they integrate with threshold control or affect equilibrium specification. *Consequence:* Framework disconnected from physiological reality
*Reflexes as fixed threshold mechanisms:* Spinal reflexes implement $\lambda$-specified thresholds automatically	Long-latency reflexes (50–100 ms) reverse sign with task instruction, modulate with movement phase, and align with voluntary goals rather than mechanical disturbance characteristics; reflex transmission is adjusted by presynaptic inhibition in task-dependent manner. Even modern EPH acknowledges reflex modulation but does not specify the principles governing this modulation	*Requires auxiliary assumption:* Task-dependent gain modulation via supraspinal control, effectively converting “automatic” reflexes into active feedback pathways with context-sensitive tuning. *Consequence:* EPH increasingly resembles feedback control with tunable gains—the framework it claimed to replace
*Temporal coordination from mechanical interactions:* Timing, velocity profiles, and phase relationships emerge from equilibrium shifts and plant dynamics	Bell-shaped velocity profiles, task-specific phase relationships, and structured multi-phase coordination require precise temporal structuring inconsistent with passive mechanical convergence; so-called spinal CPGs generate autonomous rhythms independent of descending commands in decerebrate preparations	*Unresolved problem:* EPH provides no mechanism for generating temporal structure of $\lambda$ shifts; appealing to CPGs does not solve this, as CPGs produce timing autonomously rather than implementing descending equilibrium commands. *Hidden homunculus:* Implicit timing controller must solve the coordination problem EPH claims to bypass
*Independent joint control:* Equilibrium points can be specified independently for each joint; synergies reduce complexity by grouping DOFs	Bi-articular muscles create obligatory mechanical coupling; interaction torques propagate across kinematic chain; UCM analysis reveals task-space rather than joint-space stabilization	*Requires auxiliary assumption:* Task-level specification with implicit solution of multi-joint coupling. *Consequence:* “Independent” joint control becomes coupled task-space control, contradicting core assumption
*Passive adaptation to external dynamics:* Spring-like muscles automatically compensate for loads and perturbations	Force field adaptation shows: (1) gradual learning over 20–100 trials, (2) generalization in extrinsic workspace coordinates rather than muscle/joint coordinates, (3) savings upon re-exposure and interference between opposing fields, (4) transfer across effectors. These patterns are consistent with learned dynamics representations and difficult to reconcile with passive mechanical compensation or simple threshold recalibration	*Fundamental incompatibility:* The trial-by-trial learning, workspace generalization structure, and context-dependent switching require mechanisms EPH does not provide. *Recent attempts:* Add iterative error-correction schemes, but these effectively implement the feedforward compensation EPH was meant to avoid
*Automatic stability from spring properties:* Stiffness emerges from muscle mechanics; stability and equilibrium are synonymous	Humans selectively modulate endpoint stiffness: anisotropic geometry, direction-dependent magnitude, task-specific modulation (high for precision, low for speed); postural vs. movement tasks require incompatible impedance settings	*Requires auxiliary assumption:* Active co-contraction control and impedance regulation. *Parsimony cost:* Adds control dimensions EPH was designed to eliminate. *Consequence:* Impedance becomes a control variable, not an emergent property
*No learning or adaptation mechanisms:* Framework specifies control for given task; learning unaddressed	Motor learning shows systematic improvements with practice, transfer across contexts, savings after washout, and context-dependent switching between learned dynamics—all require memory and update mechanisms	*Unresolved problem:* EPH provides no learning rules or adaptation mechanisms. *Modeling consequence:* Parameters must be manually retuned by experimenter for each condition, preventing prediction
*Minimal central representation:* CNS specifies equilibrium points; detailed execution handled peripherally	Motor cortex shows continuous population dynamics during preparation and execution, encoding of kinematics and dynamics, rapid perturbation responses, and persistent activity throughout well-learned movements	*Fundamental incompatibility:* Neural data contradicts “minimal representation” claim. *Modern EPH response:* Acknowledges cortical involvement in $\lambda$ specification, but this concedes the central computation EPH aimed to eliminate
*Scalability via synergies:* Multi-joint coordination simplified by muscle groupings with shared $\lambda$ values	Multi-joint EPH simulations require extensive manual parameter tuning, exhibit instabilities under perturbations, generate excessive self-motion and curved rather than straight paths, and cannot reproduce human obstacle avoidance strategies without adding artificial potential fields that violate EPH principles	*Modeling failure:* EPH does not scale beyond single-joint or highly constrained scenarios; synergy grouping reduces dimensionality but does not solve the specification problem. *Parameter proliferation:* Task-specific manual tuning required, preventing generalization and falsifying claims of parsimony

For each core assumption of EPH, we identify key empirical observations that contradict or require auxiliary assumptions beyond the framework’s original scope. The “Implications for EPH” column indicates whether each finding can be accommodated within the framework or forces its rejection or substantial revision. This analysis reveals that modern EPH formulations increasingly resemble the feedback control frameworks they were meant to replace, raising questions about theoretical parsimony and falsifiability

## Conclusion: lessons from the equilibrium point hypothesis

The equilibrium point hypothesis (EPH) has played a formative role in motor control. It helped unify posture and movement under a common lens, encouraged careful study of impedance and stability, and inspired tractable perturbation paradigms and surrogate models that continue to inform experimental and clinical work. By emphasizing muscle–reflex pathways and peripheral mechanics, EPH made difficult questions empirically accessible and shaped generations of inquiry. These are enduring contributions.

Our motivation in this critique is forward-looking. Because EPH remains influential in contemporary basic and translational research, it is important to clarify where the framework succeeds, where it is silent, and where its assumptions conflict with current evidence. Our goal is not to diminish past insights but to sharpen the field’s conceptual boundaries so that new, biologically grounded approaches can develop without talking past inherited metaphors.

In synthesizing theoretical, empirical, and computational work, we find several recurring challenges for EPH when extended beyond restricted conditions. First, EPH under-specifies control for realistic multi-joint behavior: inverse kinematics/dynamics, impedance regulation, and temporal coordination still need to be solved by an unspecified mechanism (the $\lambda$ specification problem). Second, key assumptions about muscle and reflex properties are difficult to reconcile with established force–length and force–velocity relations and with task-dependent reflex modulation. Third, behavioral data in perturbation, load adaptation, and obstacle-avoidance settings show flexible, goal-dependent corrections that are not well captured by passive convergence to referent states. Fourth, neural evidence points to continuous preparatory and online control of kinematics and dynamics, and computational implementations often require parameter tuning that limits generalization. These considerations suggest that EPH is most reliable as a narrow heuristic for quasi-static or single-joint contexts, and less adequate as a comprehensive theory of human movement.

We have contrasted EPH with optimal feedback control and impedance-based frameworks not because we consider these alternatives complete or unproblematic, but because they demonstrate that the phenomena EPH claims to explain can be accounted for through different mechanisms. Optimization-based theories and related computational frameworks that assume explicit prediction or model-based control carry their own computational and implementational challenges (Loeb 2012; Mangalam 2025a, b, c), and we do not advocate for their wholesale adoption. Our point is more fundamental: EPH does not uniquely explain the empirical phenomena attributed to it, and alternative accounts—even those with acknowledged limitations—handle key datasets more parsimoniously.

Moving forward, we advocate frameworks that keep faith with biological constraints and remain explicitly falsifiable. Explanations should integrate neural processes, muscle mechanics, and task-level function, and they should account for context dependence, redundancy, and multiscale organization across time and body segments. Where equilibrium-based intuitions are useful—for example, for interpreting impedance or designing specific perturbation protocols—they can be retained as local approximations within richer accounts.

Progress will benefit from interdisciplinary collaboration among neuroscientists, biomechanists, clinicians, and engineers, along with practices that improve cumulative science: clear operationalizations, preregistration where appropriate, adversarial collaboration, and benchmarks that test competing predictions under shared datasets and analysis pipelines. Such steps can help ensure that theoretical debate translates into empirical traction.

As the field evolves beyond the equilibrium metaphor, we recommend treating EPH as part of a broader toolbox rather than as a unifying theory. Doing so preserves its historical strengths—intuitions about impedance and experimental craftsmanship—while encouraging development of models that better capture the adaptive, distributed, and temporally structured nature of human movement. The path is more demanding than reliance on equilibrium alone, but it promises greater explanatory fidelity to how nervous systems, bodies, and environments together realize skilled action.

## Data Availability

No datasets were generated or analysed during the current study.
